# Effects of Different Dietary Protein Levels on the Growth Performance, Physicochemical Indexes, Quality, and Molecular Expression of Yellow River Carp (*Cyprinus carpio haematopterus*)

**DOI:** 10.3390/ani13071237

**Published:** 2023-04-02

**Authors:** Shihui Wang, Jingwen Tian, Xiaona Jiang, Chitao Li, Yanlong Ge, Xuesong Hu, Lei Cheng, Xiaodan Shi, Lianyu Shi, Zhiying Jia

**Affiliations:** 1 National and Local Joint Engineering Laboratory for Freshwater Fish Breeding, Heilongjiang River Fisheries Research Institute, Chinese Academy of Fishery Sciences, Harbin 150070, China; 2 Key Open Laboratory of Cold Water Fish Germplasm Resources and Breeding of Heilongjiang Province, Heilongjiang River Fisheries Research Institute, Chinese Academy of Fishery Sciences, Harbin 150070, China; 3 Key Laboratory of Freshwater Aquatic Biotechnology and Breeding, Ministry of Agriculture and Rural Affairs, Heilongjiang River Fisheries Research Institute, Chinese Academy of Fishery Sciences, Harbin 150070, China

**Keywords:** *Cyprinus carpio haematopterus*, aquaculture, quality evaluation, gene, protein

## Abstract

**Simple Summary:**

Yellow River Carp (*Cyprinus carpio haematopterus*) is a major commercial farmed species belonging to *Cyprinus carpio*, which has been cultivated widely, especially in Northern China. However, no relative literature focused on appropriate dietary protein level for large-sized *Cyprinus carpio haematopterus*. The present study investigated the effects of different dietary protein levels on the growth performance, physicochemical indexes, quality, and molecular expression of *Cyprinus carpio haematopterus* and aimed to determine which dietary protein level was most appropriate for *Cyprinus carpio haematopterus*. It was found that the optimal dietary protein level for *Cyprinus carpio haematopterus* (160.24 ± 15.56 g) is 250–280 g/kg based on different aspect analysis. This study is also helpful in laying the foundations for the breeding of new carp varieties with low dietary protein levels.

**Abstract:**

A 12-week rearing trial was carried out to estimate effects on the growth performance, physicochemical indexes, quality, and the molecular expression of Yellow River Carp (*Cyprinus carpio haematopterus*) using five practical diets, including dietary protein levels of 220, 250, 280, 310, and 340 g/kg. The results illustrated that the fish’s weight gain (WG) and specific growth rate (SGR) were significantly influenced, with an ascending dietary protein level of up to 250 g/kg (*p* < 0.05). The carp muscle contents of total saturated fatty acids (∑SFA), monounsaturated fatty acids (∑MUFA), polyunsaturated fatty acids (∑PUFA), and fatty acids (∑FA) decreased significantly with the ascending dietary protein levels, except for the 250 g/kg protein diet (*p* < 0.05). Only the glutamic acid and total essential amino acid (∑EAA) contents were significantly influenced by the ascending dietary protein levels (*p* < 0.05). The relative *GH* expression of the carp muscle significantly decreased with the increase in the dietary protein level up to 310 g/kg, and then it significantly increased (*p* < 0.05). In the intestines, the peak relative *TOR* expression was observed on the 220 g/kg protein diet, while the relative *4EBP1* expression was significantly influenced by the dietary protein level up to 250 g/kg (*p* < 0.05). In the muscle, the peak relative *TOR* and *4EBP1* expression levels were observed on the 250 g/kg protein diet. In gills, the lowest relative *Rhag*, *Rhbg*, and *Rhcg1* expression levels were observed on the 250 g/kg protein diet. Based on all of the aforementioned results, the optimal dietary protein level for *Cyprinus carpio haematopterus* (160.24 ± 15.56 g) is 250–280 g/kg.

## 1. Introduction

As mentioned in the State of the World Fisheries and Aquaculture report, aquatic food is making increasingly important contributions to food security and nutrition in the 21st century [1]. Fish account for more than 40% of the total production [1]. The contribution of fish, as a protein source, is very important for the global population [2]. Protein in feed is an essential factor for increasing the growth performance and production of fish, because protein is the major constituent of the fish body [3]. The protein levels of fish vary according to the species, body size, protein source, and other rearing conditions [4]. Due to their high price, the proteins account for a major proportion of the feed cost. Therefore, an appropriate dietary protein level not only maintains the optimal growth of fish but also reduces the feed cost [5]. Moreover, excessive protein intake from feed will increase amino acid metabolism pressure on the fish body and negatively influence the environment [6,7]. Therefore, the appropriate dietary protein level is an important measure not only for ensuring that fish are in the most suitable growth state but also for reducing costs and expanding the economic benefits.

In 2021, the annual yield of common carp (*Cyprinus carpio*) was 2.83 million tons, accounting for 10.73% of the total freshwater fish production in China [8]. The culture pattern includes ponds, reservoirs, lakes, and rice fields. Yellow River Carp (*Cyprinus carpio haematopterus*) is a major commercial farmed species belonging to *Cyprinus carpio*, which has been cultivated widely, especially in Northern China, because of its delicious flesh, high disease resistance, and elegant appearance, with golden scales and red or orange tail fins [9]. As previous studies have mentioned, the dietary protein levels decrease with the increase in the fish body size [10]. Previous studies on the dietary protein levels of *Cyprinus carpio* mainly focused on juvenile *Cyprinus carpio* with weights below 20 g [9,11] and juvenile *Cyprinus carpio haematopterus* with weights of 21.56 ± 0.46 g [12]. Song et al. [9] demonstrated that appropriate dietary hydroxyproline supplementation can promote myogenesis and collagen synthesis in the muscle of *Cyprinus carpio haematopterus*, and this ultimately improves the raw- and cooked-fillet texture characteristics. Yun et al. [12] posited that different dietary methionine supplementations can affect the growth performance and fillet texture characteristics of *Cyprinus carpio haematopterus*. However, limited information is available for large-sized *Cyprinus carpio haematopterus*. At the same time, feed enterprises excessively publicize the dietary protein contents of feeds to raise their price, which is unfavorable to industrial development. Therefore, there is an urgent need to identify an appropriate dietary protein level for large-sized *Cyprinus carpio haematopterus*. Hence, the present study investigated the effects of different dietary protein levels on the growth performance, physicochemical indexes, quality, and molecular expression of *Cyprinus carpio haematopterus* and aimed to determine which dietary protein level was most appropriate for *Cyprinus carpio haematopterus*. This study is also helpful in laying the foundations for the breeding of new carp varieties with low dietary protein levels.

## 2. Materials and Methods

### 2.1. Feed Formulation and Preparation

The feed formulation was designed according to the feed formula for common carp (GB/T 36782-2018, 2019) [13], and the proximate composition of the practical diets is specified in the Supplementary Materials, Table S1. Five different protein diets with 220, 250, 280, 310, and 340 g/kg of protein and a similar crude lipid level of 60 g/kg were fed to *Cyprinus carpio haematopterus*, respectively. Soybean meal was the main protein source used to regulate the different protein levels. Wheat meal was equally decreased as the dietary protein level increased to ensure that the available dietary energy was similar between all the formulations. The ingredients of the diets were thoroughly mixed together and used to produce 3.0 mm diameter feed pellets. All the diets were air-dried, sieved into pellets, and stored at −20 °C until use.

### 2.2. Fish Management and Feeding

Large-sized *Cyprinus carpio haematopterus* were obtained from the Kuandian Experimental Station (Dandong, China) of Heilongjiang Fisheries Research Institute, Chinese Academy of Fishery Sciences, China. Subsequently, the carp were marked with electronic labels and fed with a commercial diet containing a crude protein level of 280 g/kg twice each day during indoor acclimatization for two weeks. After that, the healthy fish (*n* = 75) with a mean initial body weight of (160.24 ± 15.56) g were randomly assigned to 15 indoor glass tanks (125 × 55 × 70 cm^3^), with each diet group containing a total of 15 individuals in 3 replicates.

The rearing trial lasted for three months, from 12 June to 12 September 2021. The carp were hand-fed four times each day with a ration of 40–50 g/kg of the fish’s body weight (05:30 a.m., 10:00 a.m., 02:00 p.m. and 05:00 p.m.). One-third of the total water volume was replaced twice each day (07:00 a.m. and 03:30 p.m.). Meanwhile, the remaining diet was removed at the time of water replacement to refresh the water. During the entire rearing period, the light was maintained in a natural photoperiod, the water temperature was 22.34 ± 0.56 °C, the dissolved oxygen was 6.87 ± 0.76, the total ammonia was 0.15 ± 0.03 mg/L, and the pH was 7.98 ± 0.23.

### 2.3. Sample Collection

Upon the termination of the feeding trial, all carp were fasted for 24 h. Subsequently, all carp were counted and weighed to determine the weight gain (WG), specific growth rate (SGR), and survival rate (SR). After the carp were anaesthetized with MS-222 (Sigma, St Louis, MO, USA, 0.1 g/L), a total of 15 carp, with 5 carp contained in each indoor glass tank in 3 replicates, were collected to obtain blood samples via tail vein bleeding, and the samples were prepared via centrifugation (3000× *g* rpm/min, 15 min, 4 °C) for hematological parameter detection. The separated plasma was stored at −80 °C until use. After blood sampling, the gills, intestines, and muscles of fifteen carp within each diet group were dissected and frozen immediately in liquid nitrogen and then stored at −80 °C until the analysis of the gene expression and intestinal enzyme activities [14]. Approximately 2 g of midgut tissues was removed and washed in 0.9% physiological saline that was pre-cooled at 4 °C. Intestinal tissue homogenates were collected and stored at −80 °C for the determination of intestinal digestive enzymes and antioxidant enzymes. Five individual muscles in each replicate were mixed to calculate the proximate composition and fatty acid profile and perform amino acid analysis.

### 2.4. The Growth Performance

The percentage of weight gain (WG, %) and specific growth rate (SGR, %/d) were calculated based on the initial body weight and final body weight. The percentage of survival rate (SR, %) was calculated based on the difference between the numbers of initial and final individuals. The formulae are presented in the following Equations (1)–(3):

(1) $$\mathrm{WG}\;(\%)=100\;\times \;\frac{W_{t}{-W}_{t-1}}{W_{t-1}}$$

(2) $$\mathrm{SGR}\;(\%/d)=100\;\times \;\frac{{\mathrm{LnW}}_{t}-{\mathrm{LnW}}_{t-1}}{D}$$

(3) $$\mathrm{SR}\;(\%)=100\;\times \;\frac{N_{F}}{N_{I}}$$

where W_t_ is the initial body weight and W_t−1_ is the final body weight; D is the interval (days) between the two sampling periods; N_F_ is the number of final individuals; and N_I_ is the number of initial individuals.

### 2.5. The Analysis of the Plasma Hematological Parameters and Enzyme Activities

All indices of the plasma hematological parameters and enzyme activities [14] were determined using commercial kits (Nanjing Jiancheng Bioengineering Institute, Nanjing, China), including alanine aminotransferase (ALT), aspartate aminotransferase (AST), globulin (GLO), alkaline phosphatase (ALP), total protein (TP), albumin (ALB), blood urea nitrogen (BUN), total cholesterol (TC), triglyceride (TG), high-density cholesterol (HDL-C), low-density cholesterol (LDL-C), uric acid (UA), total bile acid (TBA), α-amylase (α-AMS), lipase (LPS), trypsin (TPS), catalase (CAT), superoxide dismutase (SOD), and malondialdehyde (MDA).

### 2.6. Proximate Composition

The moisture of the carp muscle was measured using a vacuum freeze dryer (FD-1A-50, Biocoll, Beijing, China) at −50 °C, with vacuum freezing to constant weight. The five freeze-dried samples of each muscle were randomly selected and combined into one sample and then combined into three duplicate samples. The AOAC [15] method was used to determine the crude protein (Kjeldahl method) and ash heated at 550 °C to a constant weight. According to the GB 5009.6-2016 determination of fat in foods [16], the crude fat in the freeze-dried samples was extracted using the Soxhlet extraction method, and its content was determined. Based on the moisture, the dry weight of crude protein, crude fat, and ash were converted into the wet weight.

### 2.7. Fatty Acid Profile and Evaluation

The composition and content of carp muscle fatty acids were determined according to the peak area percentage method in the GB 5009.168-2016 determination of fatty acids in foods [17]. The results are presented as the percentage of each fatty acid with respect to the total fatty acids (%). The evaluation of the fatty acid quality was performed using the hypocholesterolemic/hypercholesterolemic ratio (h/H), index of atherogenicity (AI), and index of thrombogenicity (TI) [18], using Equations (4)–(6):

(4) $$h/H=\frac{\;\sum \left(18:1n9,\;18:1n7,\;18:2n6,\;18:3n6,\;18:3n3,\;20:3n6,\;20:4n6,\;20:5n\;3,\;22:4n6,\;22:5n3,\;22:6n3\right)}{\sum \left(14:0,\;16:0\right)}$$

(5) $$\mathrm{AI}=\frac{\;(12:0+4\;\times \;14:0+16:0)}{(\sum n\;-\;6\;\mathrm{PUFA}+\sum n\;-\;3\;\mathrm{PUFA}+\sum \mathrm{MUFA})}$$

(6) $$\mathrm{TI}=\frac{\left(14:0+16:0+18:0\right)}{(0.5\;\times \;\sum \mathrm{MUFA}+0.5\;\times \;\sum n\;-\;6\;\mathrm{PUFA}+3.0\;\times \;\sum n\;-\;3\;\mathrm{PUFA}+n\;-\;3/n\;-\;6\;\mathrm{PUFA})}$$

### 2.8. Amino Acid Analysis and Evaluation

The composition and content of carp muscle amino acids were analyzed according to the GB 5009.124-2016 method of determination of amino acids in foods [19]. The protein quality was evaluated using the amino acid score (AAS) and chemical score (CS) of essential amino acids (EAA). Meanwhile, the essential amino acid index (EAAI) was calculated. The AAS and CS were calculated in relation to a reference scoring pattern suggested by FAO/WHO/UNU [20]. The EAAI was calculated according to the equation described by Shahidi and Synowiecki [21]. The formulae are presented in the following Equations (7)–(9):

(7) $$\mathrm{AAS}=\frac{\mathrm{EAA}(g)\;\mathrm{in}\;\mathrm{tested}\;\mathrm{protein}\;(\mathrm{mg}/g\;N)\;}{\mathrm{EAA}(g)\;\mathrm{in}\;\mathrm{pattern}\;\mathrm{protein}\;(\mathrm{FAO}/\mathrm{WHO})\;}$$

(8) $$\mathrm{CS}=\frac{\mathrm{EAA}(g)\;\mathrm{in}\;\mathrm{tested}\;\mathrm{protein}\;(\mathrm{mg}/g\;N)\;}{\mathrm{EAA}(g)\;\mathrm{in}\;\mathrm{pattern}\;\mathrm{protein}\;(\mathrm{Egg})\;}$$

(9) $$\mathrm{EAAI}=100\times \sqrt[{n}]{\frac{A}{\mathrm{AE}}\times \frac{B}{\mathrm{BE}}\times .\;\ldots \;..\frac{G}{\mathrm{GE}}}$$

where A, B, … G refer to the contents of Ile, Leu, Lys, Thr, Val, sulfur-containing amino acids (Met + Cys), and aromatic amino acids (Phe + Tyr) in the tested protein (mg/g N, dry weight); AE, BE, … GE refer to the Ile, Leu, Lys, Thr, Val, sulfur-containing amino acids (Met + Cys), and aromatic amino acids (Phe + Tyr) in the egg protein pattern (mg/g N, dry weight); and n refers to the number of amino acids.

### 2.9. Molecular Expression Analysis

Trizol reagent was used to extract total RNA from the muscle and intestine samples. Subsequently, the extracted total RNA was quantified via spectrophotometry at 260/280 nm and verified using agarose gel electrophoresis. The first-strand cDNA was synthesized using the TaKaRa PrimeScript™ RT reagent Kit with the gDNA Eraser (RR047A, Bao bio engineering Co., Ltd., Dalian, China). Real-time quantitative PCR (qPCR) was conducted with the cDNA template and the TaKaRa SYBR^®^ Premix Ex Taq™ (Tli RNaseH Plus) (RR0420A, Bao bio engineering Co., Ltd., Dalian, China), following the manufacturer’s protocol. The primers are listed in the Supplementary Materials, Table S2.

### 2.10. Statistical Analysis

The results are presented as the mean values ± standard error (SE). SPSS 22.0 software (SPSS Inc, Chicago, IL, USA) was used for the statistical analysis. The normality and homogeneity of the results were checked with Levene’s test. When necessary, arcsine square root or logarithmic transformation was performed prior to analysis. A one-way analysis of variance (ANOVA) was used to determine the differences between these treatments, using Duncan’s multiple range tests. *p* < 0.05 was regarded as the level of statistical significance.

## 3. Results

### 3.1. The Growth Performance

The effects on the growth performance of carp with different dietary protein levels are shown in Table 1. The SR was 100% among these five treatments, and no mortality was observed. The FBW increased with the increase in the dietary protein level up to 280 g/kg (*p* < 0.05), whereas the WG and SGR were significantly influenced and increased with the ascending dietary protein level up to 250 g/kg, beyond which no further increase was detected (*p* < 0.05).

### 3.2. The Plasma Hematological Parameters and Enzyme Activities

The plasma hematological parameters and intestinal enzyme activities of the carp are presented in Table 2. No significant differences were observed in the contents of ALT, AST, GLO, TP, ALB, BUN, TC, TG, HDL-C, LDL-C, or TBA between any of the treatments (*p* > 0.05). A descending tendency of the ALP parameter, decreased from (281.58 ± 6.59) U/L to (115.12 ± 6.70) U/L, was detected via the increase in the dietary protein levels. Carp that were fed on the 340 g/kg protein diet presented the highest UA contents (28.89 ± 2.38 μmol/L), with a significant difference when compared with the other treatments (*p* < 0.05).

The intestinal digestive enzyme activity contents, including α-AMS, LPS, and TPS, showed a significant upward trend with the increase in the dietary protein level up to 280 g/kg, and then no further increase was detected (*p* < 0.05) (Table 3). The intestinal antioxidant enzyme activities of CAT and SOD first rose significantly with the increasing dietary protein level and then reduced when the dietary protein level increased above 280 g/kg (*p* < 0.05). Nevertheless, the MDA contents were significantly influenced, reducing with the ascending dietary protein level up to 280 g/kg, and then no further reduction was observed (*p* < 0.05).

### 3.3. Proximate Composition

The results of the proximate composition illustrated that the dietary protein levels had significant effects on the contents of moisture, crude protein, and crude lipids in the carp muscle (*p* < 0.05) but not on the ash content (*p* > 0.05) (Table 4). The protein content maintained a high level with the increasing dietary protein level, but it significantly dropped when the dietary protein was 340 g/kg. A similar tendency was also observed in the crude lipid contents, which decreased significantly when the dietary protein increased to 250 and 310 g/kg (*p* < 0.05).

### 3.4. Fatty Acids Profiles

The fatty acid compositions and contents of carp muscle with different dietary protein levels are presented in Table 5. Overall, the carp muscle contents of total saturated fatty acids (∑SFA), monounsaturated fatty acids (∑MUFA), polyunsaturated fatty acids (∑PUFA), and fatty acids (∑FA) decreased significantly with the ascending dietary protein levels, except for the 250 g/kg protein diet (*p* < 0.05). C16:0 was the dominant fatty acid within the SFA, while C18:1n9c was the most abundant fatty acid within the MUFA. C18:2n6c was the dominant fatty acid within the PUFA. A significant decrease in the ∑n-6 PUFA contents was noted for protein diets between 280 g/kg and 310 g/kg (*p* < 0.05). There was a significant decreasing tendency of the h/H parameters for the protein diets between the 280 g/kg and 310 g/kg (*p* < 0.05), whereas there was a significant increasing tendency of the AI parameter with the increasing dietary protein level (*p* < 0.05).

### 3.5. Amino Acids Analysis

Table 6 illustrates the amino acid compositions and contents of carp muscle with different dietary protein levels. Only the glutamic acid and ∑EAA contents were significantly influenced by the ascending dietary protein levels (*p* < 0.05). However, the other 22 amino acid parameters were not significantly influenced by the increase in dietary protein levels among any of the treatments (*p* > 0.05). The results of the protein quality evaluation of the essential amino acids demonstrated that the dietary protein levels had no significant effect on the AAS, CS, or EAAI (*p* > 0.05) (Table 7). Meanwhile, the approximate values of the parameters were observed with an increase in the dietary protein level.

### 3.6. Molecular Expression

#### 3.6.1. Relative *GH* Expression

**Figure 1:**
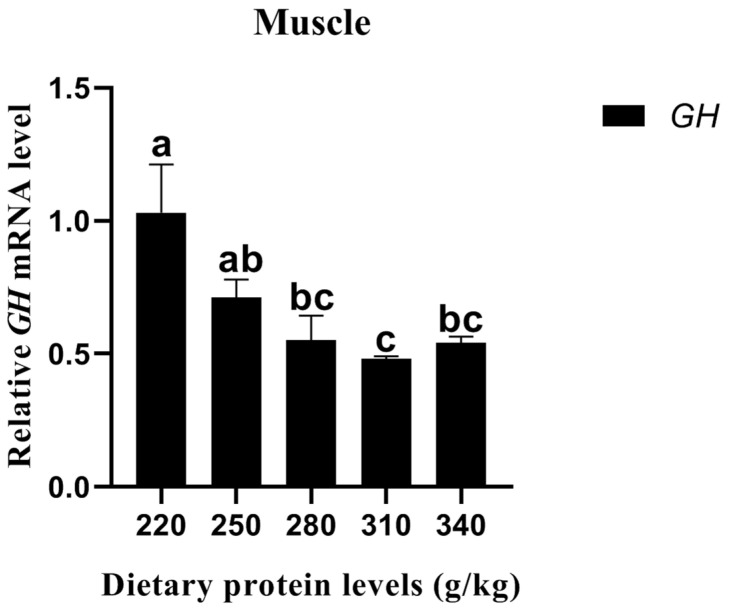
Effects of different dietary protein levels on the *GH* gene expression of *Cyprinus carpio haematopterus.* Notes: Data are presented as means ± standard error (SE). Values with different superscripts (a, b and c) are significantly different (*p* < 0.05). Abbreviations: *GH*, growth hormone.

The relative *GH* expression of carp muscle significantly decreased with the increasing dietary protein level up to 310 g/kg, and then it significantly increased (*p* < 0.05) (Figure 1). No significant difference in relative *GH* expression was detected between the 280 g/kg and 340 g/kg protein diets (*p* > 0.05).

#### 3.6.2. Relative *TOR* and *4EBP1* Expression of Protein Synthesis

**Figure 2:**
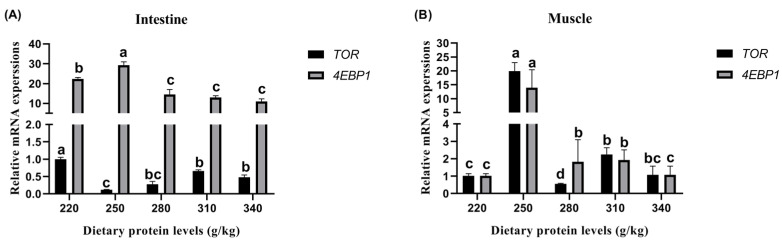
Effects of different dietary protein levels on the *TOR* and *4EBP1* gene expression of *Cyprinus carpio haematopterus*. Note: Data are presented as means ± standard error (SE). Values with different superscripts (a, b and c) are significantly different (*p* < 0.05). Abbreviations: *TOR*, target of rapamycin; *4EBP1*, eukaryotic translation initiation factor 4E binding protein 1.

The results for the relative *TOR* and *4EBP1* expression of protein synthesis in the intestines and muscle are illustrated in Figure 2. In the intestines, the peak relative *TOR* expression was observed on the 220 g/kg protein diet (Figure 2A). Similarly, the relative *4EBP1* expression was significantly influenced by the increase in the dietary protein level up to a 250 g/kg (*p* < 0.05), and then no further increase was detected among the 280 g/kg, 310 g/kg, and 340 g/kg protein diets (*p* > 0.05). In the muscle, significant changes in *TOR* and *4EBP1* expression were observed with the increase in the dietary protein level from 220 g/kg to 250 g/kg (*p* < 0.05) (Figure 2B), and then the values dropped after the dietary protein level reached beyond 280 g/kg. The peak relative *TOR* and *4EBP1* expression levels were observed in the fish on the 250 g/kg protein diet.

#### 3.6.3. Relative Rhesus Glycoprotein Expression

**Figure 3:**
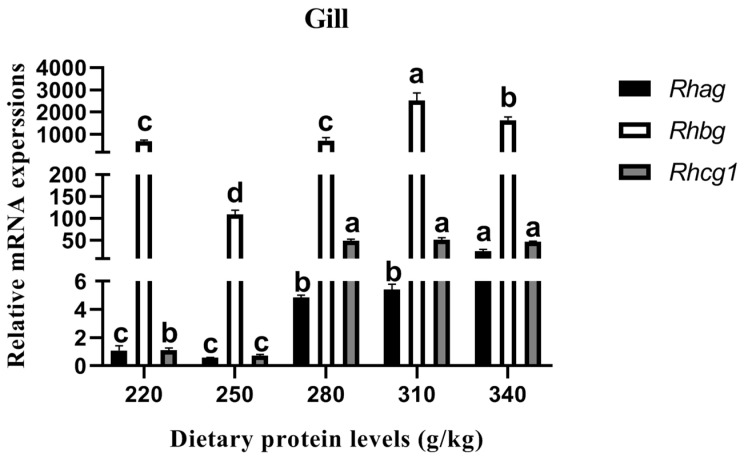
Effects of different dietary protein levels on the *RH* gene expression of *Cyprinus carpio haematopterus*. Note: Data are presented as means ± standard error (SE). Values with different superscripts (a, b and c) are significantly different (*p* < 0.05). Abbreviations: *Rhag*, Rhesus a glycoprotein; *Rhbg*, Rhesus b glycoprotein; *Rhcg1*, Rhesus c glycoprotein 1.

The relative *Rhesus glycoproteins* (*RH*) expression in the gills is shown in Figure 3. The relative *Rhag*, *Rhbg*, and *Rhcg1* expression was downregulated with the increase in the dietary protein level from 220 g/kg to 250 g/kg, albeit that no significant difference in *Rhag* between the two treatments was found (*p* > 0.05). However, significantly upregulated relative *Rhag*, *Rhbg*, and *Rhcg1* expression levels were observed with the increase in the dietary protein level from 250 g/kg to 310 g/kg (*p* < 0.05). Among these treatments, the relative *Rhag* expression on the 340 g/kg protein diet was higher than that of the other treatments (*p* < 0.05). Meanwhile, the maximum values of relative *Rhbg* and *Rhcg1* expression were detected in the fish on the 310 g/kg protein diet.

## 4. Discussion

The relatively low WG and SGR obtained in the present study were compared with the findings of published papers [14], which illustrated that there are some differences in growth performance between different carp varieties. In comparison with the same carp varieties [9,12], the IBM of large-sized *Cyprinus carpio haematopterus* was selected and found to affect the SGR and WG in the present study. The WG and SGR were first significantly enhanced with the increase in the dietary protein up to an optimal level, while thereafter, they slightly decreased, which illustrated that excessive dietary protein levels affect the nutrient utilization and feed efficiency [3], further resulting in the clear growth inhibition of fish [14]. Reductions in the WG and SGR with dietary protein levels beyond the optimal level have been observed in *Ictalurus punctatus* [22], *Mystus nemurus* [23], *Tor putitora Hamilton* [24], *Scophthalmus maximus L.* [25], *Puntius gonionotus* [26], *Pagrus* [27], and *Acipenser baerii* ♀ × *A. gueldenstaedtii* ♂ [28]. Carp may expend large amounts of energy to metabolize excess dietary protein, while the amount of energy for growth is substantially reduced, resulting in the disruption of growth [29]. Lower relative *GH* expression in the muscle may be another reason for the growth inhibition of *Cyprinus carpio haematopterus*. The present study illustrated that higher relative *GH* expression in the muscle was found in the fish on the 220–250 g/kg protein diet. A similar tendency was also observed in *Carassius auratus gibelio* [30]. The findings of the current study demonstrated that *GH* expression was significantly influenced by the ascending dietary protein level and significantly influenced the WG and SGR.

The *TOR* and *4EBP1* genes are involved in protein synthesis in the muscle and intestinal tissues of carp [14]. The present study demonstrated that a dietary protein level above 250 g/kg affected *TOR* and *4EBP1* expression and, in addition, protein synthesis. However, the crude protein and ∑TAA in the muscle were not significantly influenced by the dietary protein level of 220–310 g/kg. The aforementioned results may be explained by the following reasons: When the high protein diet was fed to *Cyprinus carpio haematopterus*, the exogenous food source of protein (their diet) was enough for the carp to accomplish the activities required for life. In contrast, when the low protein diet was fed to *Cyprinus carpio haematopterus*, the carp needed to improve their own endogenous protein synthesis in order to increase their protein deposition. Therefore, more endogenous biosynthetic pathways and proteins were promoted and accumulated via the high levels of *TOR* and *4EBP1* expression in the carp. A similar response pattern was also observed in *Epinephelus lanceolatus* [31] and *Cyprinus carpio Songpu* [14]. The ∑EAA contents showed an ascending tendency with the increase in dietary protein, indicating that the endogenous biosynthetic pathway can only synthesize the nonessential amino acids required by the carp, and the essential amino acids must be obtained via exogenous biological pathways such as the feed.

An interesting phenomenon was also noted, whereby the crude lipid and fatty acid contents of the muscle decreased significantly with the increase in dietary protein. These results were also observed in *Bidyanus bidyanus* [32], *Epinephelus coioides* [33], *Pelteobagrus fulvidraco* [34], and *Sparus macrocephalus* [35]. The important function of crude lipids is to supply energy. When the high protein diet was fed to the carp, the protein in the carp replaced the crude lipid oxidation energy supply. Therefore, the level of crude lipid accumulation gradually decreased.

For carp, one of the major functions of the gills is to excrete ammonia in active transport, which relies principally on the proteins expressed by the *RH* gene [36,37,38]. The present study demonstrated that the relative *Rhag*, *Rhbg*, and *Rhcg1* expression was significantly upregulated with increase in the dietary protein level from 250 g/kg to 310 g/kg, implying that high amounts of dietary protein increased the ammonia metabolism of the carp. These results were also observed in *Cyprinus carpio Songpu* [14]. The plasma hematological parameters and intestinal enzyme activities of carp can reflect the state of ammonia metabolism in vivo. This study showed that the carp were in a relatively poor survival state when fed with a 340 g/kg protein diet. SOD and CAT can inhibit inflammatory factors [39], scavenge free radicals [40], improve immune function [41], and perform detoxification [42] in fish. Furthermore, MDA is a typical parameter that is used to reflect the oxidative damage of the body [43]. In this study, the activities of SOD and CAT in the serum of *Cyprinus carpio haematopterus* first increased and then decreased with an increase in the dietary protein level. The content of MDA first decreased and then increased. These results are consistent with findings on *Cyprinus carpio* [44,45] and *Aristichthys nobilis* [46], implying that the antioxidant capacity of *Cyprinus carpio haematopterus* will not be weakened by a reduction in dietary protein. The present study also illustrated that the α-AMS, LPS, and TPS activities of the *Cyprinus carpio haematopterus* intestine first increased and then decreased, results which were observed in previous studies [47,48]. These results demonstrated an increase in the digestive enzyme activities of the fish intestine with the appropriate dietary protein, and excessive protein intake damaged the fish digestive function [49].

## 5. Conclusions

Based on all of the aforementioned parameters, including the WG, SGR, plasma hematological parameters, intestinal enzyme activities, proximate composition, fatty acids, amino acids, and gene expression, the optimal dietary protein level for *Cyprinus carpio haematopterus* (160.24 ± 15.56 g) is 250–280 g/kg, which helps the fish to obtain a better growth performance, physicochemical indexes, and nutritional quality. Nevertheless, excessive protein intake from the diet will place an ammonia metabolic burden on carp and increase the cost of its culture. Additionally, this study is significant for the breeding of new carp varieties with low dietary protein levels.

## Figures and Tables

**Table 1:** Effects of different dietary protein levels on the growth performance of *Cyprinus carpio haematopterus*.

Item	Dietary Protein Level (g/kg)				
	220	250	280	310	340
IBM(g)	150.44 ± 15.45	138.38 ± 9.28	175.36 ± 12.26	153.25 ± 8.76	168.32 ± 13.07
FBW(g)	269.38 ± 49.51 ^c^	309.22 ± 30.35 ^b^	364.66 ± 20.36 ^a^	305.93 ± 17.97 ^c^	297.54 ± 38.61 ^c^
WG(%)	73.30 ± 0.07 ^e^	123.46 ± 0.05 ^a^	107.94 ± 0.16 ^b^	99.63 ± 0.02 ^c^	76.77 ± 0.17 ^d^
SGR(%/d)	0.61 ± 0.02 ^e^	0.89 ± 0.01 ^a^	0.81 ± 0.01 ^b^	0.77 ± 0.08 ^c^	0.63 ± 0.05 ^d^
SR(%)	100	100	100	100	100

Notes: Data are presented as means ± standard error (SE). Values in the same row with different superscripts are significantly different (*p* < 0.05). Abbreviations: IBW, initial body weight; FBW, final body weight; WG, weight gain; SGR, specific growth rate; SR, survival rate.

**Table 2:** Effects of different dietary protein levels on the plasma hematological parameters of *Cyprinus carpio haematopterus*.

Item	Dietary Protein Level (g/kg)				
	220	250	280	310	340
ALT (U/L)	2.37 ± 0.35	2.76 ± 0.34	5.60 ± 0.36	3.00 ± 0.30	5.36 ± 0.33
AST (U/L)	21.38 ± 3.08	21.42 ± 2.34	22.98 ± 3.26	22.14 ± 3.89	31.22 ± 3.21
GLO (g/L)	18.40 ± 0.42	16.78 ± 2.86	19.32 ± 3.16	18.52 ± 0.56	17.30 ± 1.23
ALP (U/L)	281.58 ± 6.59 ^a^	236.44 ± 6.51 ^ab^	194.02 ± 7.13 ^ab^	134.46 ± 7.76 ^ab^	115.12 ± 6.70 ^b^
TP (g/L)	30.96 ± 0.89	28.06 ± 1.54	32.72 ± 1.97	31.94 ± 1.31	30.92 ± 1.70
ALB (g/L)	12.56 ± 0.56	11.28 ± 1.72	13.66 ± 1.47	13.62 ± 0.90	13.42 ± 0.69
BUN (mmol/L)	4.73 ± 0.58	5.56 ± 0.81	6.47 ± 1.08	5.66 ± 1.20	5.44 ± 1.34
TC (mmol/L)	2.84 ± 0.14	2.69 ± 0.42	2.89 ± 0.29	2.65 ± 0.11	2.47 ± 0.19
TG (mmol/L)	1.96 ± 0.26	1.51 ± 0.40	1.28 ± 0.18	1.38 ± 0.16	1.34 ± 0.26
HDL-C (mmol/L)	1.78 ± 0.08	1.68 ± 0.22	1.67 ± 0.14	1.67 ± 0.10	1.60 ± 0.09
LDL-C (mmol/L)	3.57 ± 0.14	3.35 ± 0.43	3.35 ± 0.28	3.35 ± 0.22	3.17 ± 0.18
UA (μmol/L)	7.72 ± 2.51 ^c^	23.90 ± 2.60 ^ab^	15.12 ± 3.16 ^bc^	13.40 ± 2.36 ^bc^	28.89 ± 2.38 ^a^
TBA (μmol/L)	1.52 ± 0.18	0.94 ± 0.21	1.02 ± 0.29	2.06 ± 0.76	1.72 ± 0.33

Notes: Data are presented as means ± standard error (SE). Values in the same row with different superscripts are significantly different (*p* < 0.05). Abbreviations: ALT, alanine aminotransferase; AST, aspartate aminotransferase; GLO, globulin; ALP, alkaline phosphatase; TP, total protein; ALB, albumin; BUN, blood urea nitrogen; TC, total cholesterol; TG, triglyceride; HDL-C, high-density cholesterol; LDL-C, low-density cholesterol; UA, uric acid; TBA, total bile acid.

**Table 3:** Effects of different dietary protein levels on the intestinal enzyme activities of *Cyprinus carpio haematopterus*.

Item	Dietary Protein Level (g/kg)				
	220	250	280	310	340
α-AMS(U/mgprot)	27.02 ± 4.54 ^b^	50.26 ± 6.01 ^a^	60.33 ± 4.82 ^a^	27.71 ± 6.56 ^b^	24.16 ± 2.21 ^b^
LPS (U/gprot)	8.81 ± 0.75 ^bc^	14.95 ± 2.12 ^a^	17.41 ± 0.49 ^a^	10.04 ± 0.71 ^b^	6.61 ± 0.47 ^c^
TPS (U/gprot)	5288.99 ± 135.87 ^b^	7231.77 ± 920.57 ^a^	7632.99 ± 280.69 ^a^	6295.11 ± 652.67 ^ab^	5216.29 ± 322.42 ^b^
CAT (U/mgprot)	25.84 ± 2.64 ^c^	34.50 ± 1.95 ^ab^	36.78 ± 0.92 ^a^	29.48 ± 1.39 ^bc^	29.29 ± 2.60 ^bc^
SOD (U/mgprot)	18.86 ± 0.96 ^b^	26.27 ± 1.82 ^a^	26.25 ± 1.98 ^a^	20.64 ± 2.23 ^b^	18.67 ± 0.96 ^b^
MDA (nmol/mL)	2.45 ± 0.18 ^a^	1.91 ± 0.43 ^b^	1.52 ± 0.55 ^b^	1.71 ± 0.60 ^b^	2.04 ± 0.71 ^ab^

Notes: Data are presented as means ± standard error (SE). Values in the same row with different superscripts are significantly different (*p* < 0.05). Abbreviations: α-AMS, α-amylase; LPS, lipase; TPS, Trypsin; CAT, catalase; SOD, superoxide dismutase; MDA, malondialdehyde.

**Table 4:** Effects of different dietary protein levels on the proximate composition of *Cyprinus carpio haematopterus* (%, wet weight).

Item	Dietary Protein Level (g/kg)				
	220	250	280	310	340
Moisture	77.18 ± 0.41 ^b^	79.15 ± 0.55 ^a^	78.00 ± 0.12 ^ab^	78.53 ± 1.82 ^a^	78.44 ± 0.44 ^a^
Crude protein	17.45 ± 0.28 ^a^	16.86 ± 0.16 ^a^	17.06 ± 0.43 ^a^	16.95 ± 0.43 ^a^	15.86 ± 0.38 ^b^
Crude lipid	3.14 ± 0.15 ^a^	2.24 ± 0.07 ^b^	2.97 ± 0.34 ^a^	1.46 ± 0.21 ^c^	1.96 ± 0.09 ^bc^
Ash	1.46 ± 0.07	1.29 ± 0.12	1.82 ± 0.19	1.82 ± 0.33	1.28 ± 0.04

Notes: Data are presented as means ± standard error (SE). Values with different superscripts are significantly different (*p* < 0.05).

**Table 5:** Effects of different dietary protein levels on the fatty acids and evaluation of *Cyprinus carpio haematopterus* (g/100g, dry weight).

Fatty Acids	Dietary Protein Level (g/kg)				
	220	250	280	310	340
C14:0	0.05 ± 0 ^ab^	0.03 ± 0.01 ^b^	0.05 ± 0.01 ^ab^	0.06 ± 0.01 ^a^	0.05 ± 0 ^ab^
C15:0	0.02 ± 0	0.01 ± 0	0.02 ± 0	0.02 ± 0	0.01 ± 0
C16:0	1.55 ± 0.06 ^a^	1.09 ± 0.14 ^ab^	1.36 ± 0.15 ^ab^	1.21 ± 0.22 ^ab^	0.99 ± 0.06 ^b^
C17:0	0.02 ± 0 ^a^	0.01 ± 0 ^b^	0.02 ± 0 ^a^	0.02 ± 0 ^a^	0.02 ± 0 ^a^
C18:0	0.50 ± 0.03 ^a^	0.38 ± 0.04 ^ab^	0.44 ± 0.04 ^ab^	0.41 ± 0.05 ^ab^	0.35 ± 0.01 ^b^
C20:0	0.03 ± 0 ^a^	0.02 ± 0 ^b^	0.03 ± 0 ^a^	0.02 ± 0 ^b^	0.02 ± 0 ^b^
C22:0	0.01 ± 0	0.01 ± 0	0.01 ± 0	——	——
C24:0	0.01 ± 0	0.01 ± 0	0.01 ± 0	0.01 ± 0	0.01 ± 0
∑SFA	2.19 ± 0.09 ^a^	1.56 ± 0.19 ^ab^	1.94 ± 0.21 ^ab^	1.74 ± 0.29 ^ab^	1.45 ± 0.07 ^b^
C16:1	0.13 ± 0.01	0.08 ± 0.02	0.10 ± 0.01	0.15 ± 0.04	0.11 ± 0.01
C18:1n9c	2.73 ± 0.13 ^a^	1.82 ± 0.26 ^b^	2.23 ± 0.33 ^ab^	1.97 ± 0.38 ^ab^	1.41 ± 0.06 ^b^
C20:1	0.15 ± 0 ^a^	0.09 ± 0.01 ^b^	0.13 ± 0.01 ^ab^	0.14 ± 0.02 ^ab^	0.11 ± 0.01 ^ab^
C22:1n9	0.21 ± 0.01 ^a^	0.14 ± 0.01 ^b^	0.14 ± 0.03 ^b^	0.13 ± 0.02 ^b^	0.13 ± 0.01 ^b^
C24:1	0.02 ± 0 ^a^	0.01 ± 0 ^b^	0.02 ± 0 ^a^	0.02 ± 0 ^a^	0.02 ± 0 ^a^
∑MUFA	3.23 ± 0.15 ^a^	2.15 ± 0.30 ^b^	2.62 ± 0.37 ^ab^	2.41 ± 0.43 ^ab^	1.78 ± 0.05 ^b^
C18:2n6c (LA)	3.11 ± 0.03 ^a^	2.13 ± 0.30 ^ab^	2.57 ± 0.63 ^a^	1.42 ± 0.28 ^b^	1.06 ± 0.08 ^b^
C18:3n6 (GLA)	0.04 ± 0 ^a^	0.02 ± 0 ^b^	0.02 ± 0 ^b^	0.01 ± 0 ^b^	0.01 ± 0 ^b^
C18:3n3 (ALA)	0.21 ± 0 ^a^	0.13 ± 0.02 ^ab^	0.17 ± 0.05 ^a^	0.07 ± 0.02 ^b^	0.06 ± 0.01 ^b^
C20:2	0.09 ± 0 ^a^	0.06 ± 0.01 ^ab^	0.08 ± 0.02 ^a^	0.05 ± 0.01 ^b^	0.05 ± 0 ^b^
C20:3n6	0.11 ± 0.01 ^a^	0.07 ± 0.01 ^b^	0.08 ± 0.02 ^b^	0.07 ± 0 ^b^	0.06 ± 0 ^b^
C20:3n3	0.01 ± 0 ^b^	0.01 ± 0 ^b^	0.02 ± 0 ^a^	0.01 ± 0 ^b^	0.01 ± 0 ^b^
C20:4n6 (ARA)	0.02 ± 0	0.03 ± 0.01	0.02 ± 0.01	0.01 ± 0	0.01 ± 0
C20:5n3 (EPA)	0.02 ± 0 ^b^	0.02 ± 0 ^b^	0.04 ± 0.01 ^a^	0.04 ± 0 ^a^	0.04 ± 0 ^a^
C22:6n3 (DHA)	0.16 ± 0.01	0.15 ± 0.02	0.18 ± 0.04	0.17 ± 0.03	0.19 ± 0.01
∑PUFA	3.77 ± 0.04 ^a^	2.63 ± 0.35 ^ab^	3.17 ± 0.76 ^a^	1.85 ± 0.28 ^b^	1.49 ± 0.12 ^b^
∑FA	9.19 ± 0.23 ^a^	6.34 ± 0.75 ^bc^	7.73 ± 1.11 ^ab^	5.99 ± 0.86 ^bc^	4.72 ± 0.21 ^c^
∑LC-PUFA	0.41 ± 0.01	0.34 ± 0.04	0.42 ± 0.07	0.34 ± 0.02	0.36 ± 0.02
∑HUFA	0.57 ± 0.02	0.43 ± 0.06	0.52 ± 0.12	0.38 ± 0.01	0.38 ± 0.03
∑n-3 PUFA	0.40 ± 0.01	0.32 ± 0.03	0.40 ± 0.10	0.28 ± 0.01	0.30 ± 0.02
∑n-6 PUFA	3.28 ± 0.04 ^a^	2.25 ± 0.31 ^ab^	2.69 ± 0.64 ^a^	1.51 ± 0.29 ^b^	1.15 ± 0.09 ^b^
N-3/n-6 PUFA	0.12 ± 0 ^c^	0.14 ± 0.01 ^bc^	0.15 ± 0.02 ^bc^	0.20 ± 0.04 ^ab^	0.26 ± 0 ^a^
DHA+EPA	0.18 ± 0.01	0.17 ± 0.02	0.22 ± 0.04	0.20 ± 0.03	0.23 ± 0.01
DHA/EPA	6.93 ± 0.13 ^a^	6.42 ± 0.52 ^ab^	4.79 ± 0.45 ^c^	4.57 ± 0.59 ^c^	4.96 ± 0.25 ^bc^
h/H	2.38 ± 0.06 ^a^	2.35 ± 0.03 ^a^	2.23 ± 0.34 ^a^	1.58 ± 0.2 ^b^	1.48 ± 0.04 ^b^
AI	0.25 ± 0 ^c^	0.26 ± 0 ^c^	0.28 ± 0.03 ^bc^	0.34 ± 0.03 ^ab^	0.37 ± 0.01 ^a^
TI	0.46 ± 0.01	0.45 ± 0	0.49 ± 0.07	0.55 ± 0.06	0.54 ± 0.01

Notes: Data are presented as means ± standard error (SE). “——”means no detected results. Values in the same row with different superscripts are significantly different (*p* < 0.05). Abbreviations: ∑SFA, sum of saturated fatty acids; ∑MUFA, sum of monounsaturated fatty acids; ∑PUFA, sum of polyunsaturated fatty acids; ∑FA, sum of fatty acids; ∑LC-PUFA, sum of long-chain polyunsaturated fatty acids; ∑HUFA, sum of highly unsaturated fatty acids; ∑n-3 PUFA, sum of ω-3 polyunsaturated fatty acids; ∑n-6 PUFA, sum of ω-6 polyunsaturated fatty acids; h/H, hypocholesterolemic/hypercholesterolemic ratio; AI, index of atherogenicity; TI, index of thrombogenicity.

**Table 6:** Effects of different dietary protein levels on the amino acid profile of the muscle of *Cyprinus carpio haematopterus* (g/100 g, dry weight).

Amino Acids	Dietary Protein Level (g/kg)				
	220	250	280	310	340
Valine ^#^	3.63 ± 0.44	4.21 ± 0.17	3.71 ± 0.23	4.07 ± 0.07	4.21 ± 0.06
Lysine ^#^	7.58 ± 0.08	7.68 ± 0.12	7.59 ± 0.07	7.66 ± 0.10	7.64 ± 0.14
Leucine	8.00 ± 0.36	7.50 ± 0.22	7.20 ± 0.13	7.73 ± 0.14	7.65 ± 0.27
Threonine ^#^	3.35 ± 0.32	3.90 ± 0.24	3.39 ± 0.08	3.72 ± 0.12	3.72 ± 0.28
Isoleucine ^#^	3.59 ± 0.14	3.83 ± 0.30	3.36 ± 0.03	3.70 ± 0.19	3.72 ± 0.30
Methionine ^#^	2.20 ± 0.26	2.39 ± 0.27	2.06 ± 0.16	2.09 ± 0.22	2.47 ± 0.05
Phenylalanine ^#^	3.09 ± 0.09	3.09 ± 0.09	3.10 ± 0.10	3.11 ± 0.11	3.10 ± 0.10
∑EAA	31.44 ± 0.38 ^ab^	32.60 ± 0.88 ^a^	30.41 ± 0.34 ^b^	32.07 ± 0.36 ^ab^	32.51 ± 0.85 ^a^
Alanine *	4.07 ± 0.24	4.36 ± 0.14	4.71 ± 0.23	4.48 ± 0.29	4.30 ± 0.11
Glycine *	3.20 ± 0.03	3.22 ± 0.01	3.20 ± 0.04	3.23 ± 0.03	3.20 ± 0.01
Aspartic acid *	8.77 ± 0.07	8.62 ± 0.23	8.79 ± 0.01	8.87 ± 0.03	8.84 ± 0.09
Glutamic acid *	12.22 ± 0.01 ^a^	12.21 ± 0 ^ab^	12.16 ± 0.02 ^b^	12.18 ± 0.03 ^ab^	12.22 ± 0 ^ab^
∑UAA	28.26 ± 0.19	28.41 ± 0.34	28.87 ± 0.19	28.76 ± 0.31	28.56 ± 0.13
Arginine	4.24 ± 0.39	4.26 ± 0.35	4.40 ± 0.09	4.99 ± 0.17	5.07 ± 0.23
Cysteine	0.82 ± 0.06	0.84 ± 0.02	1.03 ± 0.11	0.81 ± 0.07	0.81 ± 0.04
Histidine	2.10 ± 0.06	2.13 ± 0.07	2.10 ± 0.06	2.11 ± 0.06	2.13 ± 0.08
Proline	2.79 ± 0.32	2.46 ± 0.26	2.66 ± 0.21	2.46 ± 0.26	2.11 ± 0.06
Serine	3.29 ± 0.31	3.85 ± 0.34	3.43 ± 0.03	3.78 ± 0.21	3.73 ± 0.32
Tyrosine	1.72 ± 0.19	1.80 ± 0.08	1.40 ± 0.20	1.67 ± 0.31	1.45 ± 0.24
∑NEAA	43.22 ± 1.27	43.75 ± 0.89	43.89 ± 0.83	44.58 ± 1.22	43.86 ± 0.75
∑TAA	74.66 ± 1.65	76.35 ± 1.27	74.31 ± 0.71	76.65 ± 1.51	76.37 ± 1.58
EAA/TAA (%)	42.13 ± 0.41	42.69 ± 0.81	40.94 ± 0.65	41.86 ± 0.50	42.56 ± 0.26
UAA/TAA (%)	37.88 ± 0.72	37.23 ± 0.77	38.86 ± 0.30	37.53 ± 0.40	37.42 ± 0.68
EAA/NEAA (%)	212.32 ± 13.44	213.16 ± 8.83	203.34 ± 10.6	203.92 ± 10.59	212.76 ± 3.74

Notes: ^#^ is an essential amino acid. * means umami amino acid. Values in the same row with different superscripts are significantly different (*p* < 0.05). Abbreviations: ∑EAA, total essential amino acids; ∑UAA, total umami amino acids; ∑NEAA, total nonessential amino acids; ∑TAA, total amino acids; EAA/TAA, percentage of ∑EAA to ∑TAA; UAA/TAA, percentage of ∑UAA to ∑TAA; EAA/NEAA, percentage of ∑EAA to ∑NEAA.

**Table 7:** Effects of different dietary protein levels on the protein quality evaluation of essential amino acids in *Cyprinus carpio haematopterus*.

Essential Amino Acids	FAO/ WHO	Egg Protein	AAS					CS				
			220	250	280	310	340	220	250	280	310	340
Isoleucine	2.50	3.31	0.90 ± 0.04	0.96 ± 0.08	0.84 ± 0.01	0.92 ± 0.05	0.93 ± 0.07	0.72 ± 0.03	0.77 ± 0.06	0.68 ± 0.01	0.74 ± 0.04	0.75 ± 0.06
Leucine	4.40	5.34	1.14 ± 0.05	1.07 ± 0.03	1.02 ± 0.02	1.10 ± 0.02	1.09 ± 0.04	0.94 ± 0.04	0.88 ± 0.03	0.84 ± 0.01	0.90 ± 0.02	0.90 ± 0.03
Lysine	3.40	4.41	1.39 ± 0.02	1.41 ± 0.02	1.40 ± 0.01	1.41 ± 0.02	1.40 ± 0.03	1.07 ± 0.01	1.09 ± 0.02	1.08 ± 0.01	1.09 ± 0.01	1.08 ± 0.02
Threonine	2.50	2.92	0.84 ± 0.08	0.97 ± 0.06	0.85 ± 0.02	0.93 ± 0.03	0.93 ± 0.07	0.72 ± 0.07	0.83 ± 0.05	0.73 ± 0.02	0.80 ± 0.03	0.80 ± 0.06
Valine	3.10	4.10	0.73 ± 0.09	0.85 ± 0.04	0.75 ± 0.05	0.82 ± 0.01	0.85 ± 0.01	0.55 ± 0.07	0.64 ± 0.03	0.56 ± 0.03	0.62 ± 0.01	0.64 ± 0.01
Methionine + Cysteine	2.20	3.86	0.86 ± 0.07	0.92 ± 0.08	0.88 ± 0.07	0.82 ± 0.07	0.93 ± 0.02	0.49 ± 0.04	0.52 ± 0.04	0.50 ± 0.04	0.47 ± 0.04	0.53 ± 0.01
Phenylalanine + Tyrosine	3.80	5.65	0.79 ± 0.04	0.80 ± 0.02	0.74 ± 0.02	0.79 ± 0.03	0.75 ± 0.04	0.53 ± 0.03	0.54 ± 0.02	0.50 ± 0.01	0.53 ± 0.02	0.50 ± 0.03
EAAI								68.76 ± 1.72	73.00 ± 2.34	67.23 ± 0.95	70.77 ± 2.02	71.78 ± 2.25

## Data Availability

The original contributions presented in the study are included in the article/Supplementary Materials. Further inquiries can be directed to the corresponding authors.

## Supplementary Materials

The following supporting information can be downloaded at: https://www.mdpi.com/article/10.3390/ani13071237/s1, Table S1: Formulation and proximate composition of the experimental diets (g/kg, dry matter); Table S2: The sequences of primers used in real-time quantitative PCR analysis.
