# Supplementary material for: Effects of Different Dietary Protein Levels on the Growth Performance, Physicochemical Indexes, Quality, and Molecular Expression of Yellow River Carp (Cyprinus carpio haematopterus)
Source: Animals (Basel). 2023 Apr 2;13(7):1237. doi: 10.3390/ani13071237 (PMC10093604; doi:10.3390/ani13071237)
Supplement: Supplementary file 1 [file animals-13-01237-s001.zip › animals-2253138-supplementary.pdf]

Supplementary Materials

Table S1

Formulation and proximate composition of the experimental diets (g/kg, dry matter)

Table S2

The sequences of primers used in Real-time quantitative PCR analysis.

Table S1

Formulation and proximate composition of the experimental diets (g/kg, dry matter).

Ingredient	Dietary protein level (g/kg)				
	**220**	**250**	**280**	**310**	**340**
Soybean meal	12.52	16.37	26.81	30.00	30.00
Wheat meal	60.00	56.58	46.12	35.30	23.28
Cottonseed meal	2.00	2.00	2.00	9.48	15.00
Rapeseed meal	13.23	5.00	5.00	5.00	11.35
Fish meal	2.00	10.00	10.00	10.00	10.00
Soybean oil	4.25	4.05	4.08	4.22	4.37
Zeolite	2.00	2.00	2.00	2.00	2.00
Bentonite	2.00	2.00	2.00	2.00	2.00
$Ca(H_2PO_4)_2$	1.00	1.00	1.00	1.00	1.00
Premix	1.00	1.00	1.00	1.00	1.00
**Proximate composition (g/kg, wet matter basis)**					
Crude protein	220.2	250.7	281.0	311.0	340.9
Crude lipid	60.5	60.2	60.8	60.6	60.9

Table S2

The sequences of primers used in Real-time quantitative PCR analysis.

Gene	Sequences of primers (5′-3′)
GH	F:ATCTTCCCTCTGTCTTTCTGC<br>R:AAGTCGGCCAGCTTCTCA
TOR	F: CCACAACGCAGCCAACAA<br>R:GCCACAGAATAGCAACCCT
4EBP1	F:GCTACCTCACGACTATTGC<br>R:TTCTTGCTTGTCACTCCTG
Rhag	F:AGCAGTGTGGGCATCAACCT<br>R:CTCCAGCAGGGTCATGATGAG
Rhbg	F:ATGCAGGGCTTTTTCCACGG<br>R: TGGATCCGCCAGCATCATTG
Rhcg1	F: AGTCTGACACTCGCTGGATCG<br>R: AGCAGAGCCCACTGAATTCC
β-actin	F:GGCAGGTCATCACCATCGG<br>R: TTGGCATACAGGTCTTTACGG
